# The impact of spectacle correction on the well-being of children with vision impairment due to uncorrected refractive error: a systematic review

**DOI:** 10.1186/s12889-023-16484-z

**Published:** 2023-08-18

**Authors:** Govender-Poonsamy Pirindhavellie, Ai Chee Yong, Khathutshelo Percy Mashige, Kovin S. Naidoo, Ving Fai Chan

**Affiliations:** 1 Present Address: University of KwaZulu Natal, College of Health Sciences, Private Bag X54001, Durban, 4000 South Africa; 2 Queens University Belfast, University Rd, Belfast, BT7 1NN UK

**Keywords:** Vision impairment, Well-being, Uncorrected refractive error, Spectacle correction, Children

## Abstract

**Background:**

Despite being easily corrected with eyeglasses, over two-thirds of the world’s child population presents with vision impairment (VI) due to uncorrected refractive errors. While systematic reviews have shown that VI can significantly impact children’s depression and anxiety, none have reviewed the existing literature on the association between spectacle correction and well-being. This review aims to address this knowledge gap.

**Main outcome measures:**

The main outcome measures were i) cognitive and education well-being which included mathematics and english literacy, reading fluency, school function, academic performance and grades; ii) psychological and mental health well-being which included physical anxiety, learning anxiety and mental health test scores and iii) quality of life.

**Methods:**

We searched eight databases for articles published between 1999 to 2021 that assessed the associations between spectacle correction and children’s (0 to 18 years) well-being. There were no restrictions on language or geographic location. Two reviewers independently screened all publications using validated quality checklists. The findings of the review were analysed using narrative synthesis. [PROSPERO CRD42020196847].

**Results:**

Of 692 records found in the databases, six randomised control trials, one cohort, one cross-sectional and one qualitative study (*N *= 9, 1.3%) were eligible for analysis. Data were collected from 25 522 children, 20 parents and 25 teachers across the nine studies. Seven were rated as good quality (67 to 100% of quality criteria fulfilled), and two were satisfactory (33 to 66% of quality criteria fulfilled). Spectacle correction was found to improve children’s educational well-being (*n* = 4 very strong evidence; *n* = 2 strong evidence), quality of life (*n* = 1, very strong evidence) and decrease anxiety and increase mental health scores (*n* = 1, strong evidence).

**Conclusion:**

Evidence suggests that spectacle correction improves children’s cognitive and educational well-being, psychological well-being, mental health, and quality of life. More research is needed, given the paucity of published literature and the focus on only three aspects of well-being.

**Supplementary Information:**

The online version contains supplementary material available at 10.1186/s12889-023-16484-z.

## Background

It has been estimated that 19 million children have vision impairment, with uncorrected refractive error (URE) affecting 12 million children [1], making it the leading cause of childhood vision impairment worldwide [2]. Although spectacles can effectively correct refractive errors, less than one-third of children who need spectacles in low-resource settings have them [3]. Studies have found that vision impairment and ocular morbidities have increased anxiety [4], reading difficulties [5–7], anti-social behaviour [8], quality of life issues [9] and problems with self-esteem [10].

A meta-analysis by Mavi et al. found that children with uncorrected hyperopia had lower education performance (Standardised Mean Difference [SMD] − 0.18, 95%CI − 0.27 to − 0.09) and reading skills (SMD − 0.46, 95% CI, − 0.90 to − 0.03) compared to emmetropic children [11]. Another systematic review by Li and Chan et al. found that vision-impaired children have higher depression (SMD 0.57, 95%CI 0.26–0.89) and anxiety scores (SMD 0.61, 95% CI 0.40–0.82) than normally sighted children. The same systematic review also observed myopic children having higher depression scores than normally sighted children (SMD 0.59, 95% CI 0.36–0.81) [12]. Uncorrected myopia is also shown to impact children’s mental health negatively [13]. Furthermore, many of these studies were cross-sectional [7–10, 13–16] and could not demonstrate the causal relationship between VI due to URE and aspects of well-being due to the inherent limitations of the study design.

Studies on the impact of spectacle correction on children’s well-being are rare, with most focusing on the impact on academic performance [11, 17–20]. However, child well-being is a multi-dimensional construct that explores various domains of a child’s life, including health, education, living conditions, material well-being, and interpersonal relations [21]. The scarcity of conclusive evidence makes promoting healthy well-being in children by correcting their refractive error challenging despite having a long-term impact on their later years and into adulthood, affecting their ability to actively participate in society, their communities, and their families [22].

No systematic review of the impact of spectacle correction on the broader aspects of children’s well-being has been published or registered to date. Considering the increasing evidence on the detrimental effects of vision impairment due to URE on children, we conducted a review to systematically synthesise the findings from the existing literature on the impact of spectacle correction on various aspects of children’s well-being.

## Methods

This systematic review was registered on the International Prospective Register of Systematic Reviews (PROSPERO Registration number: CRD42020196847). The Preferred Reporting Items for Systematic Reviews and Meta-Analyses for Systematic Review Protocols (PRISMA-P) guidelines were used to develop and report the systematic review protocol [23].

The search strategy was adapted to each electronic database, including MEDLINE, PubMed, Embase, SCOPUS, ProQuest, Weipu Database (VIP), Chinese National Knowledge Infrastructure (CNKI) and Wanfang databases using the search terms in Supplementary file 1. The inclusion criteria were as follows: studies of any language from any geographic locations, published between 1999 and 2021, qualitative, observational or interventional studies; participants were children 18 years and younger diagnosed with vision impairment due to uncorrected refractive error, without any ocular comorbidities. No grey literature was included in the review. The intervention of interest was spectacle correction. The primary outcome was well-being according to the Organisation of Economic and Co-operative Development’s [24] Measuring What Matters for Child Well-being and Policies, which includes social health, physical health, cognitive and education and material health, in addition to physiological and mental health and quality of life.

Two reviewers (PGP and ACY) independently screened all titles and abstracts against eligibility criteria. If an article’s eligibility could not be determined based on its title and abstract, its full text was retrieved and screened by the two same reviewers. A third senior reviewer (VFC) was consulted in cases of disagreement between the two reviewers. Data were then extracted into a pre-designed Microsoft Excel Spreadsheet, capturing information on the publication date, author(s), title, geographic location, study setting, study design, sample size, sampling method, outcome measure, and key findings, limitations, strengths and recommendations. Two data extractors (PGP and ACY) checked for data errors and consistencies.

Two reviewers (PGP and ACY) independently appraised the quality of each randomised control trial (RCT) and qualitative study using the Critical Appraisal Skills Programme checklists (CASP) [25]. In contrast, cohort and cross-sectional studies were appraised using the Joanna Briggs Institute’s Critical Appraisal-Checklists (JBI) [26]. Discrepancies were resolved through discussion and consensus. A third senior reviewer (VFC) was consulted in cases of disagreement between the two reviewers.

Due to the high heterogeneity nature of the studies, we performed narrative synthesis on the findings to report the wide range of study design, characteristics, and well-being outcomes. The quality of the studies was reported as per the criteria of the appraisal tools. The quality of the study evidence was rated as Good (fulfilled 67 to 100% of criteria), Satisfactory (fulfilled 33 to 66% of criteria) or Poor (fulfilled 0 to 33% of criteria) [27].

## Results

**Fig. 1:**
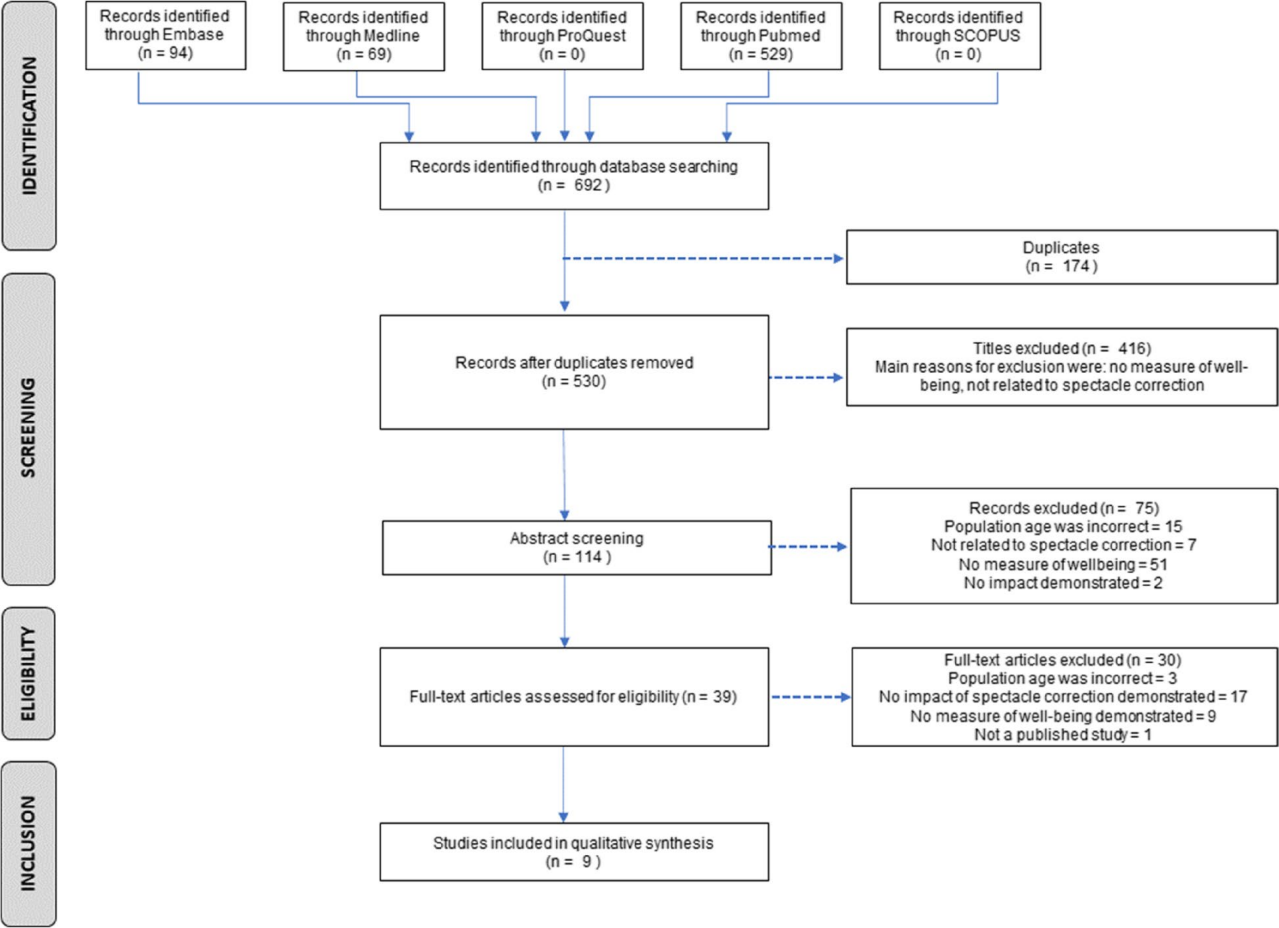
Flow chart of results of the search strategy and study selection processes

The electronic database search yielded 692 studies. After removing 174 duplicates and 416 studies due to irrelevant titles, 102 studies were included for abstract screening. Seventy-five studies were excluded. Of the 39 studies that underwent full-text screening, 30 (76.9%) were excluded because i) they did not demonstrate the impact of spectacle correction directly or as a comparison to before the spectacle correction was used (*n* = 17; 43.6%) [28–44], ii) they did not measure the impact of spectacles on children’s well-being (*n* = 9; 23.1%) [45–53], iii) the population’s age was older than 18 years-old (*n* = 3; 7.7%) [54–56] and iv) it was not a published study (*n* = 1; 2.6%) [57]. Nine full-text studies were eligible for data extraction and appraisal [17, 18, 20, 58–63]. The study selection processes are outlined in the PRISMA flow depicted in Fig. 1.

### Characteristics of the eligible studies

The eligible studies included six randomised control trials [20, 58, 59, 61–63], one cohort study [18], one cross-sectional study [60] and one qualitative study [60] published between 2012 and 2020. Five were conducted in China [20, 58, 59, 61, 63], three in the United States of America (19–21) and one in the Netherlands [62] (Table 1). Data was gathered from 25 522 children, 20 parents and 25 teachers across the nine reviewed studies.

**Table 1:** Description of the study characteristics, population, key findings, study limitations, strengths and recommendations for studies reporting the educational impact of spectacle correction (*n* = 7)

Authors/ year of publication/ Study design	Population (age range or grade level) at study enrolment/ Geographic Location/ Country Profile/ Study setting	Intervention/Type of refractive error corrected	Comparison	Tools and methods used	OUTCOME/KEY FINDINGS	Sampling Method	Sample Size	Limitations	Strengths	Recommendations
Hannum and Zhang / 2012 / RCT [59]	9–12 years old / China / LMIC / Rural	Spectacle correction / Not specified	Children who did not receive spectacle correction	– Literacy assessment – A curriculum-based math achievement test – A curriculum-based language achievement test	Spectacle correction had a significant favourable effect on math and literacy performance and class failure: – Literacy outcome: effect was 0.34 SD (95% CI: 0.130–0.557) – Math achievement outcome: effect is 0.26 SD (95% CI: 0.052–0.462) – Language achievement outcome: the effect was not significant – Children who received glasses from this project were 35% less likely to fail a class (p < 0.01)	Randomisation at the township level	19,185	It was uncertain if the strategy for matching the treatment and control groups could account for pertinent differences in confounding variables	– The randomised nature of the trial by count – The use of curriculum-based achievement tests	The selectivity issues in the study suggest that further empirical studies are needed to test the impact of vision correction on learning outcomes
Van Rijn / 2014 / RCT [62]	9–10 years old / Netherlands / HIC / Urban	Spectacle correction / Hyperopia	Differing magnitudes of refractive correction	Reading speeed tests: – Klepel (reading speed of nonwords) – The One-Minute Test (reading speed of genuine words)	Differing impact of spectacle correction on reading speed using the Klepel and one-minute reading speed tests was recorded for subjects myopia and hyperopia: – Myopes showed a small (2.44, CI: 0.39–4.49) and significant (p = 0.021) difference from baseline to follow-up with the Klepel test; Analysis of the impact of spectacle correction on reading speed amongst hyperopes found: – Hyperopes with full prescription showed a significant (p = 0.012) increase (8.77, CI: 1.59–15.93) compared to no correction and the group receiving 0.50 prescription (8.40, CI: 1.09–15.71; p = 0.019)	Randomisation of subjects in the treatment arm	191	– The small sample size did not allow unequivocal conclusions to be made – The instantaneous measurement of reading speed did not allow an assessment of the frequency of spectacle wear and its impact – There was no masking between the groups receiving and not receiving spectacle correction – A large number of subjects were lost to follow-up	– The subjects recruited for this study were those who did not plan to receive a prescription	A larger study is recommended with larger sample size
Harvey et al / 2016 [60] / Cross-sectional	Grade 3–8 / USA / HIC / Urban	Spectacle correction /Astigmatism	Differing levels of astigmatism	– Oral Reading Fluency test: Dynamic Indicators of Basic Early Literacy Skills (DIBELS)	This cross-sectional study found that: – Spectacle correction resulted in improved oral reading fluency (ORF) in subjects with astigmatism o High astigmats showed a mean improvement in ORF of 6.05 words per minute with spectacle correction o Moderate astigmats showed a mean improvement in ORF of 1.87 words per minute with spectacle correction	Purposive sampling of astigmatic students	273	– Use of grade level rather than age across groups – Absence of data on the effects of text size and near acuity on ORF in the sample – The study did not measure the impact of spectacle correction over time due to its cross-sectional nature	– Large school-based sample of bilateral astigmats with with-the-rule astigmatism – A comparison group of students with low or no astigmatism from the same cohort – Use of a well-validated tool to measure Oral Reading Fluency	– Individuals with high astigmatism should adhere to full-time spectacle wear
Dudovitz et al / 2016 [17] / Qualitative	5–14 years old / USA / HIC / Urban	Spectacle correction / Not specified	None	– Focus Group Interviews	This qualitative study investigated the relationship between vision care, students’ academic performance, classroom behaviour and psychosocial well-being using focus groups with students, their families and teachers The study found the following: – Obtaining glasses resulted in improved school function due to: o Better grades and academic performance o Improved reading ability o Increased willingness to practice academic skills and o More accuracy in math-related homework – Less disruptive behaviour in the classroom resulted in an enhanced learning environment – Greater ease in completing homework	Convenience	21	– The qualitative study nature may result in an over-representation of specific participant contributions – Aggregate group data may not reflect the specific concerns of each participant within the group – Convenience sampling has likely caused selection bias, limiting generalisation to all parents, teachers, and children served by the VTL programme	– Forging stronger partnerships between education and health sectors, schools serve as a vehicle for health service delivery and a platform for changing social and cultural norms – Results from this study can inform school and health policy for visual screening and referral services for students	– Forging stronger partnerships between education and health sectors, schools serve as a vehicle for health service delivery and a platform for changing social and cultural norms
Ma et al / 2018 / RCT [61]	10–12 years old / China / LMIC / Rural	Spectacle correction / Not specified	Late referral group	– A standardised Mathematics test	Early provision of spectacles improved children’s academic performance – Effect on mathematics scores was 0.25 SD (95% CI: 0.01–0.48; p = 0.04)	Cluster Randomisation	949	– All schools were taken from one county in rural northwest China, thereby limiting external validity – The unadjusted effect size of the main study outcome was not statistically significant – A modest follow-up of 79%	– The randomised design of the study – The use of population-based sampling	The calculation of program costs and economic modelling was recommended for the future
Nie et al / 2019 [20] / RCT	Grade 7–8 / China / LMIC / Rural	Spectacle correction / Myopia	Children receiving a prescription to purchase spectacles	– Standardised maths exam – Aspiration of further schooling – School dropout behaviour	This RCT which explored the impact of free spectacles on student’s math scores, aspirations for further schooling and dropout behaviour, found: – Improved math scores by approximately 0.14 SD; – raised aspirations to attend academic high school by nine percentage points; – reduced dropout by 44% during the school year Subjects without spectacles at baseline displayed the greatest increase in math scores of 0.196 SD than those with spectacles at baseline	Cluster Randomisation	979	– The study was conducted in one poor region of western China, and therefore findings may not be applied to other settings Effects on the dropout and other study outcomes could only be examined over the school year, making it possible that the intervention delayed students’ decisions to leave school until after the end of the school year	– The results of the study adds to the body of knowledge on the effect of spectacle provision on student academic performance	The subsidisation of spectacles for myopic students in China and other developing countries
Dudovitz et al / 2020 [18] / Cohort	Elementary school / USA / HIC / Urban	Spectacle correction / Not specified	Non-participating same grade peers who did not receive glasses	– Achievement marks in Mathematics, English language Arts (ELA) – Work habits – Behaviour rank	This cohort study which investigated grades in ELA and Mathematics, work habits and behaviour, found the following after receiving spectacles: – Subjects showed significant, improved ELA achievement rank in the fifth grading period of 5.07 (P = .001) and the sixth grading period of 3.38 PP (*p* = 0.03) – While there was no significant change in overall math achievement, subjects performing in the lower tercile showed an immediate and sustained improvement of 10 to 24 PP from baseline – There was no significant improvement in work habits over the two years follow-up period – There was a decrease in behaviour rank during the fourth grading period of 3.9 PP (*p* = 0.01), which returned to baseline levels during the fifth and sixth grading periods	Purposive sampling of VTL students	406	– The observational study design makes it impossible to attribute causation to improved school performance – Using class rank rather than grades or test scores allowed the researchers to account for school-level differences. However, it prevented classroom-level differences – It was likely that were unmeasured differences between participants and non-participants – Since the sample was mainly low-income Latino gathered from a single, large, urban school district. The results were more likely not generalisable to other populations	The study provides a quantitative assessment of whether school-based vision care improves grades among US elementary students	– Future studies to explore potential differences by VI diagnosis, age or grade level and socio-demographics

*RCT*  Randomised control trial, *RE*  Refractive error, *USA* United States of America, *LMIC* Low to Middle Income Country, *HIC*   High income country, *TG*Treatment group, *CG*  Control group, *VTL*  Vision To Learn, *CI* Confidence Intervals , *SD* Standard Deviations, *PP* Percentile Points, *CI* Confidence Interval

Seven studies investigated children’s cognitive and educational well-being, where four were RCTs [20, 59, 61, 62], one cohort [18], one cross-sectional [60] and one qualitative study [17]. The remaining two studies in this review explored the impact of spectacle correction on children’s psychological and mental health well-being [58] and children’s quality of life [63]. Randomisation was adopted in six of the nine studies either by township or cluster [3, 20, 59], by study participants [62, 63] or schools [58]. Two studies used purposive sampling [18, 60], and one study used convenience sampling [17].

The studies investigating the impact of spectacle correction on cognitive and educational well-being used a range of indicators, with Ma et al. [61] using only math scores; Hannum and Zhang [59] using math scores, literacy and language achievement tests; Dudovitz et al. [18] using achievement marks in math, English language arts, work habits and behaviour; Nie et al.[20] using math scores, aspirations for further schooling and school dropout rate; the studies by van Rijn et al. [62] and Harvey et al. [60] used reading speed of words and nonwords and oral reading fluency respectively. The RCT by Guan et al. [58] was the only study that explored the impact of myopic spectacle correction on children’s mental health using the validated Mental Health Test (MHT) score and its subscales of learning and physical anxiety scores. Zhou et al. [63] investigated quality of life using the validated National Eye Institute Refractive Error Quality of Life-42 (NEI-RQL-42) questionnaire. The qualitative study by Dudovitz et al. [17] used focus group interviews to explore parent, teachers and student perspectives on how spectacle correction improves child well-being and school function.

### The impact of spectacle correction on children’s cognitive and educational well-being

Trials by Ma et al. [61], Nie et al. [20], and Hannum and Zhang [59] that assessed math scores as an outcome found improvement by 0.25 standard deviations (SD) (satisfactory quality evidence), 0.14 SD (good quality evidence) and 0.26 SD (good quality evidence) in math scores post spectacle correction. Dudovitz et al. [18] found that after the provision of spectacle correction, students who performed in the bottom tercile for math rank at baseline achieved a significant immediate and sustained improvement of 10 to 24 percentile points (p < 0.001) (good quality evidence).

In addition to math scores, Hannum and Zhang’s [59] trial also found an average treatment effect of 0.34 SD on literacy assessment (good quality evidence) and a 44% reduction in the chances of failing a class (*p* < 0.01). Dudovits et al. [17] found 4.5 percentile points improvement (*p* = 0.02) for English Language Arts (good quality evidence) post-correction. Harvey et al. [60] also found a mean significant improvement in oral reading frequency of 6.05 words per minute (*p* = 0.001) among moderate astigmats and an improvement of 1.87 words per minute (*p* = 0.193) with spectacle correction (good quality evidence).

The qualitative study by Dudovitz et al. [17] found that providing corrective lenses to children improved their school function (good quality evidence), including behaviour or focus, willingness to practise academic skills, and improved academic performance. Participants reported improved reading ability due to a greater willingness to practice academic skills, more accuracy with math-related homework, effort and task persistence, improved concentration and focus in the classroom, less disruptive behaviour in the classroom, and more engagement and ability to participate in class (good quality evidence). A minor theme noted in this study was the ease with which homework was executed after receiving glasses.

### The psychological and mental health impact of spectacle correction

Guan et al. [58] found a significant decline (0.08 SD; *p* < 0.10) in physical anxiety among children post myopic correction (good quality evidence). There was no significant impact on learning anxiety or overall mental health (good quality evidence). The study also found spectacle correction significantly improved the MHT score (0.26 SD; *p* < 0.05) in students studying at a high intensity (more than two hours a day), but those who studied at a moderate degree of intensity (between half an hour to two hours a day) experienced a decline in MHT score (0.13SD; *p* = 0.03) (good quality evidence). Students studying at a low-intensity level (studying for less than half an hour per day) experienced an increase in Learning Anxiety (0.17SD; *p* < 0.05) (good quality evidence). Albeit insignificant, children also experienced a decline in learning anxiety (0.25 SD, *p* < 0.10); a decrease in physical anxiety (0.22 SD; *p* < 0.10) post-correction (good quality evidence).

### Quality of life impact of spectacle correction

Zhou et al. [63] found that irrespective of the method of refraction used to determine the spectacle correction, all methods showed a significant increase in quality-of-life scores with spectacle correction ranging from 2.32 [95% CI (0.37, 4.27) *p* = 0.020] in the group tested by an optometrist to 4.65 [95% CI (2.45, 6.86) *p *< 0.001] in the group receiving ready-made spectacles (good quality evidence).

Tables 1, 2 and 3 show that irrespective of the aspect of well-being investigated, spectacle correction had a positive impact, improving well-being. However, the effect of confounding factors on the study results was unclear in most cases In four out of nine studies, the type of refractive error being corrected was not specified [17, 18, 59, 61], three studies used myopic correction, [20, 58, 63] one used hyperopic correction [62], and one study used astigmatic correction [60].

**Table 2:** Description of the study characteristics, population, key findings, study limitations, strengths and recommendations for studies reporting the psychological impact of spectacle correction (*n* = 1)

Authors/ year of publication/ Study design	Population (age range or grade level) at study enrolment / Geographic Location / Country Profile / Study setting	Intervention / Type of refractive error corrected		Comparison	Tools and methods used	OUTCOME/KEY FINDINGS	Sampling Method	Sample Size	Limitations	Strengths	Recommendations
Guan et al / (2018) / RCT [58]	9–12 years old / China / LMIC / Rural		Spectacle correction / Myopia	Students who received only prescriptions	– Mental Health Test (MHT) with special attention to learning anxiety and physical anxiety	The impact of providing fully subsidised glasses on mental health resulted in the following: – When considering the total sample, glasses resulted in a 0.08 decrease in physical anxiety (p < 0.1) Sub-group analysis revealed the following: – Students who studied at a low-intensity level (studying for less than half an hour per day) experienced a 0.17SD increase in Learning Anxiety (p < 0.05) – Students who studied at a high-intensity level (studying more than two hours per day) experienced: o 0.25 SD decline in Learning Anxiety (p < 0.1); o 0.22 SD decline in Physical Anxiety (p < 0.1); o 0.26 SD improvement in MHT score (p < 0.05) – Students who studied at a moderate degree of intensity (studying between half an hour to two hours per day) experienced a 0.13 decline in MHT score (p < 0.05)	Randomisation of schools to TG and CG	2851	– Average treatment recorded could have masked heterogenous effects	– Large sample size Randomised study design	– Need to boost wear rates of glasses among students – Care must be taken to eliminate teasing of students who are newly wearing glasses Spectacles should be promoted as a learning asset among children

*RCT* Randomised control trial,* RE* Refractive error,* LMIC* Low to Middle Income Country*, TG* Treatment group,* CG* Control group, *SD *Standard Deviations

**Table 3:** Description of the study characteristics, population, key findings, study limitations, strengths and recommendations for studies reporting the quality of life impact of spectacle correction (*n* = 1)

Authors/ year of publication/ Study design	Population (age range or grade level) at study enrolment/ Geographic Location/Country Profile/Study setting	Intervention/Type of refractive error corrected	Comparison	Tools and methods used	OUTCOME/KEY FINDINGS	Sampling Method	Sample Size	Limitations	Strengths	Recommendations
Zhou et al. / (2016) / RCT	12–15 years old / China / LMIC/ Rural	Spectacle correction/ Myopia	Differing spectacle prescriptions determined by an optometrist, self-refraction, rural refractionist and ready-made spectacles	– National Eye Institute Refractive Error Quality of Life-42 (NEI-RQL-42) questionnaire	The National Eye Institute Refractive Error Quality of Life questionnaire assessing the visual function-related quality of life showed increases in scores from the baseline assessment of: – 2.32 [95% CI (0.37, 4.27) *p* = 0.020] in the group tested by an optometrist – 4.65 [95% CI (2.45, 6.86) p < 0.001] in the group receiving ready-made spectacles – 4.13 [95% CI (2.04, 6.23) p < 0.001] in the group tested by a rural refractionist – 3.14 [95% CI (1.05, 5.23) p = 0.004] in the self-refraction group Irrespective of the type of spectacles or method of correction, all findings reveal an increase in quality-of-life scores with correction	Randomisation of subjects to TG (3 groups) and CG	542	– The enrolled schools were selected using non-random sampling – All schools were drawn from a single region in southern China, thereby limiting the application of findings from the study to other contexts	– The study followed a randomised controlled design – The study had a high follow-up rate	– Further research is needed to assess the acceptability of adjustable spectacles for actual wear by adults and children

*RCT* Randomised control trial,* RE* Refractive error,* LMIC*  Low to Middle Income Country,* TG* Treatment group,* CG* *Control group*, *CI *Confidence Interval

### Quality appraisal of studies

The results of the quality appraisal are summarised in Table 4. Seven of the nine studies (77.8%) were rated as good quality, and two (22.2%) were satisfactory [27]. All studies in the review addressed a focused issue. Among the RCTs, randomisation of the groups to either the intervention or control groups was done at the cluster or school levels to ensure that participants were blind to the interventions. However, this made blinding of investigators in these clusters or schools difficult as they could easily see which groups were provided with the interventions. Almost all RCTs (*n* = 4, 67.6%) did not accurately report the data; only one (16.67%) could not generalise findings beyond the study areas. In the cohort study by Dudovitz et al. [18] it was unclear if the exposure and outcomes were measured in a valid or reliable way and if the loss to follow-up was explored. It was also unclear if confounding factors were identified in the cross-sectional study to assess the impact of spectacle correction on reading fluency [60].

**Table 4:** The checklist results for assessing the methodological quality of the included studies

**Randomised control trials**	**Aspect of Well-being**	**Q1**	**Q2**	**Q3**	**Q4**	**Q5**	**Q6**	**Q7**	**Q8**	**Q9**	**Q10**	**Q11**	**Quality % rating**
Guan et al 2018 [58]	Psychological and mental health	✓	✓	✓	±	✓	x	✓	x	x	x	✓	**54.5**
Hannum & Zhang 2012 [59]	Cognitive and education	✓	✓	✓	✓	✓	✓	✓	x	x	✓	✓	**81.8**
Ma et al 2018 [61]	Cognitive and education	✓	✓	✓	±	✓	✓	✓	x	x	✓	✓	**72.7**
Nie et al 2019 [20]	Cognitive and education	✓	✓	✓	±	✓	✓	✓	x	✓	✓	✓	**81.8**
Van Rijn 2014 [62]	Cognitive and education	✓	✓	✓	x	✓	✓	✓	✓	x	x	✓	**72.7**
Zhou et al 2016 [63]	Quality of life	✓	✓	✓	✓	✓	✓	✓	✓	x	✓	✓	**90.9**
**Cohort study**		**Q1**	**Q2**	**Q3**	**Q4**	**Q5**	**Q6**	**Q7**	**Q8**	**Q9**	**Q10**	**Q11**	
Dudovitz et al. 2020 [18]	Cognitive and education	✓	✓	±	✓	✓	✓	±	✓	±	±	✓	**63.6**
**Cross-sectional study**		**Q1**	**Q2**	**Q3**	**Q4**	**Q5**	**Q6**	**Q7**	**Q8**				
Harvey et al 2016 [60]	Cognitive and education	✓	✓	✓	✓	±	✓	✓	✓				**87.5**
**Qualitative study**		**Q1**	**Q2**	**Q3**	**Q4**	**Q5**	**Q6**	**Q7**	**Q8**	**Q9**	**Q10**		
Dudovitz 2016 [17]	Cognitive and education	✓	✓	✓	✓	✓	±	✓	✓	✓	✓		**90.0**

Question key: *CASP tool questions for randomised controlled trials assessment:* Q1 = “Did the trial address a clearly focused issue?”, Q2 = “Was the assignment of patients to treatments randomised?”, Q3 = “Were all of the patients who entered the trial properly accounted for at its conclusion?”, ? = "Is it worth continuing?", Q4 = "Were patients, health workers and study personnel 'blind' to treatment?", Q5 = "Were the groups similar at the start of the trial?", Q6 = "Aside from the experimental intervention, were the groups treated equally?", Q7 = "How large was the treatment effect?", Q8 = "How precise was the estimate of the treatment effect?", Q9 = "Can the results be applied to the local population, or in your context?", Q10 = "Were all clinically important outcomes considered?", Q11 = "Are the benefits worth the harms and costs?" [25]

*JBI tool questions for cohort study assessment:* Q1 = “Were the two groups similar and recruited from the same population?”, Q2 = “Were the exposures measured similarly to assign people to both exposed and unexposed groups?”, “Is it worth continuing?”, Q3 = “Was the exposure measured in a valid and reliable way?” Q4 = “Were confounding factors identified?”, Q5 = “Were strategies to deal with confounding factors stated?”, Q6 = “Were the groups/participants free of the outcome at the start of the study (or at the moment of exposure)?”, Q7 = “Were the outcomes measured in a valid and reliable way?”, Q8 = “Was the follow up time reported and sufficient to be long enough for outcomes to occur?”, Q9 = “Was follow up complete, and if not, were the reasons to loss to follow up described and explored?”, Q10 = “Were strategies to address incomplete follow up utilised?”, Q11 = “Was appropriate statistical analysis used?” [26]

*CASP tool questions for qualitative studies assessment:* Q1 = "Was there a clear statement of the aims of the research?", Q2 = "Is a qualitative methodology appropriate?", "Is it worth continuing?", Q3 = "Was the research design appropriate to address the aims of the research?", Q4 = "Was the recruitment strategy appropriate to the aims of the research?", Q5 = "Was the data collected in a way that addressed the research issue?", Q6 = "Has the relationship between researcher and participants been adequately considered?", Q7 = "Have ethical issues been taken into consideration?", Q8 = "Was the data analysis sufficiently rigorous?", Q9 = "Is there a clear statement of findings?", Q10 = "How valuable is the research?" [25]

*JBI tool questions for cross-sectional studies assessment:* Q1 = "Were the criteria for inclusion in the sample clearly defined?", Q2 = "Were the study subjects and the setting described in detail?" = "Is it worth continuing?", Q3 = "Was the exposure measured in a valid and reliable way?", Q4 = "Were objective, standard criteria used for measurement of the condition?", Q5 = "Were confounding factors identified?", Q6 = "Were strategies to deal with confounding factors stated?", Q7 = "Were the outcomes measured in a valid and reliable way?", Q8 = "Was appropriate statistical analysis used?" [26]

*Answers legend:* =  ✓yes the study satisfactorily met the respective quality criterion; = xno the study did not meet the respective quality criterion; ±  = can’t tell or unclear whether the study met the respective quality criterion

Scoring of quality: Yes was given a score of 1, and no or cannot tell scored as zero (0). The total score was given as a percentage of the score for each study over the total number of criteria for quality [27]

*Rating of quality of the studies*: Good = study fulfils 67 to 100% of criteria, Satisfactory = study fulfils 33 to 66% of criteria, Poor = study fulfils 0 to 33% of criteria (Dhirar et al*.*, 2020)

## Discussion

This systematic review summarised the literature narratively on the impact of spectacle correction on well-being in children with VI due to uncorrected refractive error. All the eight eligible studies were of good or satisfactory quality. The limited evidence suggests that spectacle correction improves children’s cognitive and education, psychological and mental health well-being and quality of life.

Even though well-being is multi-dimensional and comprises a range of aspects, [64] the review found seven studies that focused strongly on cognitive and educational well-being, with limited studies (*n* = 1) on psychological and mental health and quality of life (*n *= 1). School is a significant part of a child’s life [65], and many learning activities are visually based [66], including reading, digital media and observation, thereby creating a dependency on optimal visual function [67]. Therefore, combining the fact that academic performance is a key predictor of lifelong health [68], and the availability of standardised testing could also lead to the focus on educational impact.

Overall, there was good quality [18, 20, 59, 60, 63] and satisfactory quality evidence [17, 61, 62] that spectacle correction can improve cognitive and educational well-being. Nie et al. [20], Ma et al. [61], and Hannum and Zhang’s [59] trials and, Dudovits et al.’ s [18] cohort study, has sufficient power to strongly suggest that spectacle correction can improve mathematic scores. Most of these studies were conducted in China and may limit the ability to apply their findings to other contexts. However, seeing almost half of the children in the world with VI due to URE live in Asia [69], with almost 80% of myopic adolescents living in East Asia having distance vision impairment, these findings are critically relevant in this geographic location. The scarcity of evidence outside of China also highlighted the opportunities for further research in other parts of the world.

Furthermore, despite Ma et al.’ s [61] argument that change in mathematics scores is a more valid and sensitive outcome for spectacle correction, other studies also suggest improvement in literacy, English, and reading tests. Wang et al. [70] suggested that poor academic performance in subjects such as math and English could be attributed to reduced capacity for children to perform optimally on visually demanding tasks in a modern classroom. The quantitative findings were also supported by the focus group findings from Dudovits et al.’ s [17] qualitative research that explained the perceived causal pathway of improving school function. Qualitative research on this topic is rare, but it allows a deeper understanding of experiences, phenomena, and context and explains the quantitative findings to understand human experience.

Children were at a higher risk of developing poor vision by spending more time on learning [70], and increased near work due to academic pressures has also been implicated in the increase in refractive error, particularly myopia [71]. Hence, it is not surprising that while our review shows that the positive impact of spectacle correction on psychological and mental health well-being was found among Chinese children, the greatest effect was found among children who studied for longer periods in the day [58]. We hypothesise that poorer vision prior to correction is likely to yield a greater perceived impact of the correction on the visually demanding tasks associated with the academic environment.

Numerous studies investigate the impact of uncorrected refractive error and vision impairment on aspects of quality of life in children, finding the decreased quality of life in individuals with vision impairment or uncorrected refractive error [9, 72–75]. Furthermore, numerous tools quantitatively assess the refractive error-related quality of life [76]. However, there is a paucity of studies investigating the impact of spectacle correction on the quality of life in children. The study by Zhou et al. [63] provides good evidence that quality-of-life scores increase with spectacle correction. The strength of this study is noted in the use of a self-reported measure of the quality of life (NEI-RQL-42) recommended over parental proxy reporting [77]. However, Kaphle emphasised that refractive correction may not address all quality of life issues related to URE.

While spectacle correction provides a convenient and, in many cases, cost-effective method of refractive correction, it is met with poor compliance, in many cases attributed to stigma and misconceptions [27]. Our review can be used to allay these misconceptions and educate spectacles users ahead of time as it clearly shows the positive impact of spectacle correction on academic performance, mental health and quality of life.

The limitations of this review must be acknowledged. One, the restriction to only published studies which have excluded unpublished reports, such as grey literature and programme evaluations, often provide a wealth of information that published studies do not capture [78]. These evaluation reports usually focus on the implementation effectiveness and provide a wealth of information on effective spectacle provision implementation strategies but often, lack methodological rigour to capture the impact of spectacle correction on children’s well-being. Future research could strive to incorporate these valuable sources of information without compromising on rigor by using an effectiveness-implementation hybrid designs in the programme evaluation. Two, due to the mutlifaceted factors contributing to uncorrected refractive error which could significantly impact the outcomes of spectacle correction, it reduces the generalizability of the results. These factors may include physical factors (inadequate access to routine eye examinations or a lack of trained eye care professionals, especially in rural or underserved areas, leading to a lack of diagnosis) and geographical, socioeconomic, and cultural factors (geographical constraints, and lack of awareness about the importance of spectacle correction can impede access to corrective measures). Consequently, future research should aim to capture these varied contexts and factors to allow comprehensive understanding of the impacts of spectacle correction, ensuring that the benefits observed can be appropriately generalised and applied to various settings and populations. Three, we also did not conduct a meta-analysis in this review due to the great variation in study methodologies and outcomes.

## Conclusion

This review found that the limited studies touched the surface of the complex well-being construct regarding the impact of spectacle correction on children. The findings suggest that children have improved cognitive and educational well-being, psychological and mental health well-being and quality of life. More research is needed in different geographical locations to explore the impact of spectacle correction on the wider array of well-being constructs.

### Supplementary Information

**Additional file 1.**

## Data Availability

The study protocol can be accessed on PROSPERO (42,020,196,847). Additional data not presented in the manuscript can be obtained from the authors by reasonable request.

## Abbreviations

CI: Confidence interval
RCT: Randomised control trial
VI: Vision impairment
URE: Uncorrected refractive error
SMD: Standaridised mean difference
