# Supplementary material for: The impact of spectacle correction on the well-being of children with vision impairment due to uncorrected refractive error: a systematic review
Source: BMC Public Health. 2023 Aug 18;23:1575. doi: 10.1186/s12889-023-16484-z (PMC10436410; doi:10.1186/s12889-023-16484-z)
Supplement: Supplementary file 1 — Additional file 1. [file 12889_2023_16484_MOESM1_ESM.docx]

Supplementary material

Table 1. Reasons for exclusion after full-text screening

| **No.** | **Year** | **Authors** | **Title of Publication** | **Reason for Exclusion** |
| --- | --- | --- | --- | --- |
|  | 2019 | Black, et al. | In-school eyecare in special education settings has measurable benefits for children's vision and behaviour. | No measure of well-being |
|  | 2015 | Bogdănici, et al. | Quality of life for amblyopic children and their parents. | No measure of well-being |
|  | 2006 | Castanon-Holguin | Factors Associated with Spectacle-Wear Compliance in School-Aged Mexican Children | Not related to impact of spectacle correction |
|  | 2005 | Dias, et al. | Evaluating the self-esteem of myopic children over a three-year period: The COMET experience. | Not related to impact of spectacle correction |
|  | 2007 | Esteso, et al. | Correction of moderate myopia is associated with improvement in self-reported visual functioning among Mexican school-aged children. | No measure of well-being |
|  | 2018 | Evans, et al. | Vision screening for correctable visual acuity deficits in school-age children and adolescents. | No measure of well-being |
|  | 2021 | Gao et al. | The impact of spectacle lenses for myopia control on visual functions. | Population age incorrect |
|  | 2006 | Glewwe et al. | the impact of eyeglasses on the academic performance of primary school students: evidence from a randomised trial in rural China | Not a published study |
|  | 2013 | Gogate, et al. | Spectacle compliance amongst rural secondary school children in Pune district, India. | Not related to impact of spectacle correction |
|  | 2016 | Gogate, et al. | Impact of correcting visual impairment and low vision in deaf-mute students in Pune, India. | No measure of well-being |
|  | 2010 | Jones-Jordan, et al. | A comparison of spectacle and contact lens wearing times in the ACHIEVE study. | Not related to impact of spectacle correction |
|  | 2010 | Li et al. | Investigation of the psychological health of first-year high school students with myopia in Guangzhou. | Not related to impact of spectacle correction |
|  | 2014 | Ma, et al. | Safety of Spectacles for Children's Vision: A Cluster-Randomized Controlled Trial. | Not related to impact of spectacle correction |
|  | 2011 | Martin, et al. | Psychological well-being in visually impaired and unimpaired individuals: A meta-analysis | Not related to impact of spectacle correction |
|  | 2006 | Monteiro, et al. | Use of optical aids by visually impaired students: social and cultural factors. | Not related to impact of spectacle correction |
|  | 2019 | Morjaria, et al. | Predictors of Spectacle Wear and Reasons for Nonwear in Students Randomised to Ready-made or Custom-made Spectacles: Results of Secondary Objectives From a Randomised Noninferiority Trial. | No measure of well-being |
|  | 2008 | Nanthavisit, et al. | Survey of refractive errors among Buddhist scripture, Dhamma-Bali and regular school of Buddhist novices in the Bangkok metropolitan area. | No measure of well-being |
|  | 2008 | Odedra, et al. | Barriers to spectacle use in Tanzanian secondary school students. | Not related to impact of spectacle correction |
|  | 2001 | Orfield et al. | Vision problems of children in poverty in an urban school clinic: their epidemic numbers, impact on learning, and approaches to remediation. | Not related to impact of spectacle correction |
|  | 2018 | Qian, et al. | Spectacles utilisation and its impact on health-related quality of life among rural Chinese adolescents. | Not related to impact of spectacle correction |
|  | 2012 | Queirós, et al. | Quality of life of myopic subjects with different methods of visual correction using the NEI RQL-42 questionnaire. | Population age incorrect |
|  | 2006 | Robaei, et al. | Refractive Error and Patterns of Spectacle Use in 12-Year-Old Australian Children. | Not related to impact of spectacle correction |
|  | 2010 | Schneider, et al. | Frequency, Course, and Impact of Correctable Visual Impairment (Uncorrected Refractive Error). | No measure of well-being |
|  | 2012 | Sharma, et al. | School-based approaches to the correction of refractive error in children. | No measure of well-being |
|  | 2006 | Vaughn et al. | The association between vision quality of life and academics as measured by the College of Optometrists in Vision Development Quality of Life questionnaire. | Not related to impact of spectacle correction |
|  | 2014 | von-Bischhoffshausen et al. | Spectacle-wear compliance in school children in Concepción Chile. | Not related to impact of spectacle correction |
|  | 2006 | Walline, et al. | The adolescent and child health initiative to encourage vision empowerment (ACHIEVE) study design and baseline data. | Not related to impact of spectacle correction |
|  | 2020 | Wang, et al. | A Randomised Noninferiority Trial of Wearing Adjustable Glasses versus Standard and Ready-made Spectacles among Chinese Schoolchildren: Wearability and Evaluation of Adjustable Refraction III. | Not related to impact of spectacle correction |
|  | 2012 | Wills, et al. | Effect of simulated astigmatic refractive error on reading performance in the young. | Population age incorrect |
|  | 2015 | Yi, et al. | Impact of Free Glasses and a Teacher Incentive on Children’s Use of Eyeglasses: A Cluster-Randomized Controlled Trial | Not related to impact of spectacle correction |
